# A Novel Fabricating Method of Micro Lens-on-Lens Arrays with Two Focal Lengths

**DOI:** 10.3390/mi12111372

**Published:** 2021-11-08

**Authors:** Xin Liu, Min Li, Jiang Bian, Junfeng Du, Bincheng Li, Bin Fan

**Affiliations:** 1 Institute of Optics and Electronics, Chinese Academy of Sciences, Chengdu 610209, China; limin414@mails.ucas.ac.cn (M.L.); bianjiang007@126.com (J.B.); dujunfeng@ioe.ac.cn (J.D.); 2 School of Optoelectronic Science and Engineering, University of Electronic Science and Technology of China, Chengdu 611731, China; 3 University of Chinese Academy of Sciences, Beijing 100049, China

**Keywords:** microlens arrays, micro lens-on-lens arrays, photoresist reflow method, soft lithography, nano-imprint technology, mask alignment exposure technology

## Abstract

Micro lens-on-lens array (MLLA) is a novel 3D structure with unique optical properties that cannot be fabricated accurately and quickly by existing processing methods. In this paper, a new fabricating method of MLLAs with two focal lengths is proposed. By introducing the soft lithography technology, nano-imprint technology and mask alignment exposure technology, MLLAs with high precisions can be obtained. A MLLA is successfully fabricated with two focal lengths of 58 μm and 344 μm, and an experiment is carried out. The results show that the MLLA has excellent two-level focusing and imaging abilities. Furthermore, the fabricated profiles of the MLLA agree well with the designed profiles, and the morphology deviation of the MLLA is better than 2%, satisfying the application requirements. The results verify the feasibility and validity of the novel fabricating method. By adjusting mask patterns and processing parameters, MLLAs with both changeable sizes and focal lengths can be obtained.

## 1. Introduction

In recent years, microlens arrays have been widely used in the fields of optical sensing technology, laser beam shaping, artificial compound eye structures, optical fiber coupling, light-filed cameras, micro-manufacturing and lab-on-a-chip systems [1,2,3,4,5,6,7]. Micro lens-on-lens arrays (MLLAs) with multiple focal lengths have great potential in the fields of real-time detection of unconfined or fluctuating targets, 3D reconstructions and imaging in multiple depth of fields [8,9,10].

The commonly used processing methods of traditional microlens arrays are the photoresist reflow method [11,12,13], gray scale lithography [14,15,16], photolithographic ion-exchange technology [17,18], etc. However, all of these traditional methods have to be improved for the fabrication of MLLAs due to their complex structures, increasing the processing difficulties. In 2015, YANG et al. [19] first proposed a two-step femtosecond laser wet etching method to fabricate MLLAs. By adjusting the laser power and etching time, they obtained MLLAs with both changeable sizes and focal lengths. However, the surface roughness of the MLLA structure is large, with a deviation of 5%, and the image qualities are not good enough, due to blurred edges of the images. Recently, Cao A et al. [20,21] employed moving mask exposure technology to fabricate MLLAs. The mask pattern was converted into a two dimensional pattern with adjustable exposure time. The MLLA was fabricated by moving the mask with a uniform speed and by controlling the exposure time with a high precision. The problem with this method is the undesired processing accuracy. Furthermore, this method cannot be used to fabricate MLLAs with small apertures. There are two reasons for this. The first reason is that a gap that is larger than 20 μm has to be given to avoid friction and the effect of the flatness between the mask and photoresist, making the minimum line width after exposure larger than 4 μm. The second reason is that the exposure resolution can be affected by other processing parameters, such as the critical dimension and the linear precision of the moving mask. All of these factors lead to large resolution values.

This paper proposes a novel approach to fabricate MLLAs based on the traditional photoresist reflow method. MLLAs with high precisions can be obtained by introducing soft lithography technology, nano-imprint technology and mask alignment exposure technology. By adjusting mask patterns and processing parameters, MLLAs with both changeable sizes and focal lengths can be acquired.

## 2. Materials and Methods

**Figure 1:**
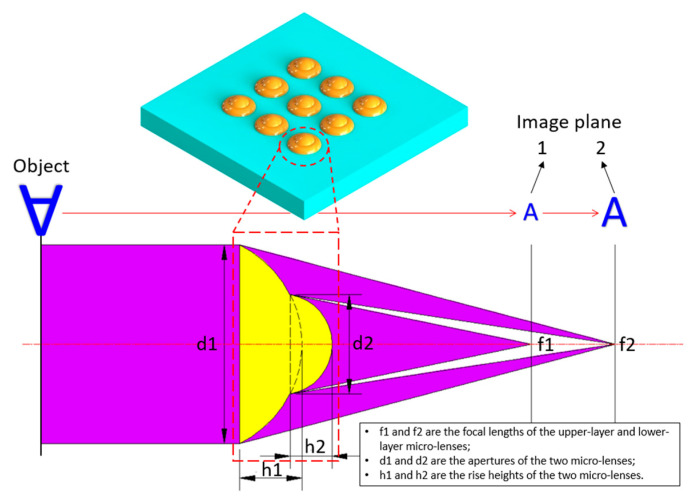
Focusing ability of the MLLAs.

The structure of MLLAs is shown as Figure 1. Each element is composed of two microlenses. The upper-layer microlens with a smaller size is fabricated on the lower-layer microlens with a larger size, and both microlenses share the same optical axis. The shapes of the two microlenses are standard spheres due to the effect of surface tension. The radiuses of the two lenses can be calculated according to Equation (1).

(1) $$\left\{ \begin{array}{l} r_{1}=\frac{d_{1}{}^{2}+4h_{1}{}^{2}}{8h_{1}}\approx \frac{d_{1}{}^{2}}{8h_{1}}\\ r_{2}=\frac{d_{2}{}^{2}+4h_{2}{}^{2}}{8h_{2}}\approx \frac{d_{2}{}^{2}}{8h_{2}} \end{array}\right.$$

where, *r*_1_ and *r*_2_ are the radiuses of the upper-layer and lower-layer microlenses. *d*_1_ and *d*_2_ are the apertures of the two microlenses. *h*_1_ and *h*_2_ are the rise heights of the two lenses.

The morphology of the MLLAs can be calculated using Equation (2).

(2) $$h(r)=\{\begin{array}{c} \sqrt{r_{1}{}^{2}-r^{2}}-(r_{1}-h_{1}),\frac{d_{2}}{2}\leq \left|r\right|\leq \frac{d_{1}}{2}\\ \sqrt{r_{2}{}^{2}-r^{2}}-(r_{2}-h_{2})+\sqrt{r_{1}{}^{2}-{\left(\frac{d_{2}}{2}\right)}^{2}}-(r_{1}-h_{1}),\left|r\right|<\frac{d_{2}}{2} \end{array}$$

The focal lengths of the upper and lower lenses are defined as Equation (3).

(3) $$\left\{ \begin{array}{l} f_{1}=\frac{r_{1}}{n-1}=\frac{d_{1}{}^{2}}{8h_{1}\left(n-1\right)}\\ f_{2}=\frac{r_{2}}{n-1}=\frac{d_{2}{}^{2}}{8h_{2}\left(n-1\right)} \end{array}\right.$$

where, *f*_1_ is the focal length of the upper-layer microlens and *f*_2_ is the focal length of the lower-layer microlens. *n* is the refractive index of the material.

Usually, MLLAs have two different focal lengths. Inverted and reduced image arrays can be obtained on the two focal planes of the MLLAs. Additionally, multiple high-resolution images can be achieved if multiple targets are placed in the range of the depth of field of the MLLAs.

**Figure 2:**
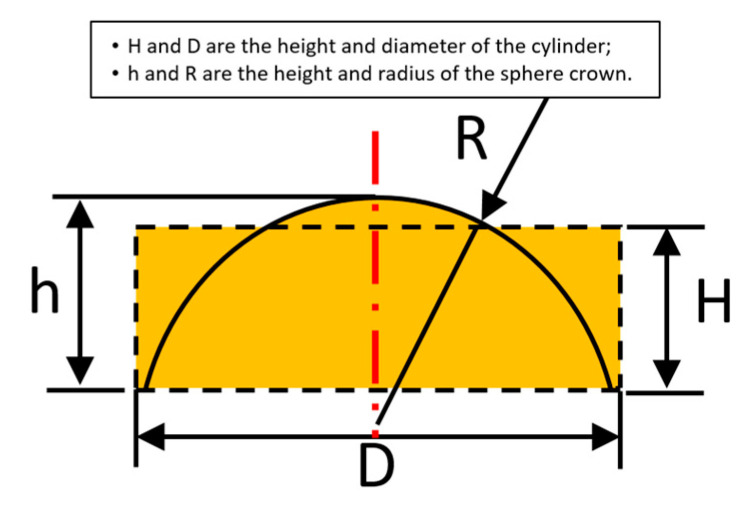
Structures of photoresist before and after melting.

The principle of the traditional photoresist reflow method is as follows. Photoresist cylindrical arrays with a height of *H* and a width of *D* are heated to a molten state, and the cylindrical structure becomes a sphere with a height of *h* and a radius of *R* due to the effect of surface tension. The structures of the photoresist before and after melting are shown in Figure 2. Compared with the initial volume of the photoresist (V1), the volume after melting (V2) reduces for the reason that some of the organic solvents in the photoresist evaporate. The percentages of organic solvents are different for the photoresist with variable version and viscosity. Assuming that the scaling factor is k, volumes V1 and V2 satisfy the relationship V2 = k*V1. The structural parameters of MLLAs can be calculated according to Equation (4).

(4) $$\left\{ \begin{array}{l} \pi h^{2}R-\frac{\pi }{3}h^{3}=k\cdot \pi {\left(\frac{D}{2}\right)}^{2}H\\ f=\frac{R}{n-1}=\frac{D^{2}}{8h\left(n-1\right)}\\ F\#=\frac{f}{D} \end{array}\right.$$

where, *R*, *f* and *F#* are parameters of the microlens array, according to which the structural parameters (*D* and *H*) of cylinders before melting can be calculated. *f* is the focal length of the microlens, and *F#* is the *F* number. Then, the mask patterns can be designed, and the version as well as the thickness of the photoresist can be determined.

For the traditional photoresist reflow method, the two-layer coaxial photoresist cylinders with different apertures become one microlens when they are melted. As a result, this method can only be applied to fabricate one-layer microlens arrays, instead of the MLLAs.

**Figure 3:**
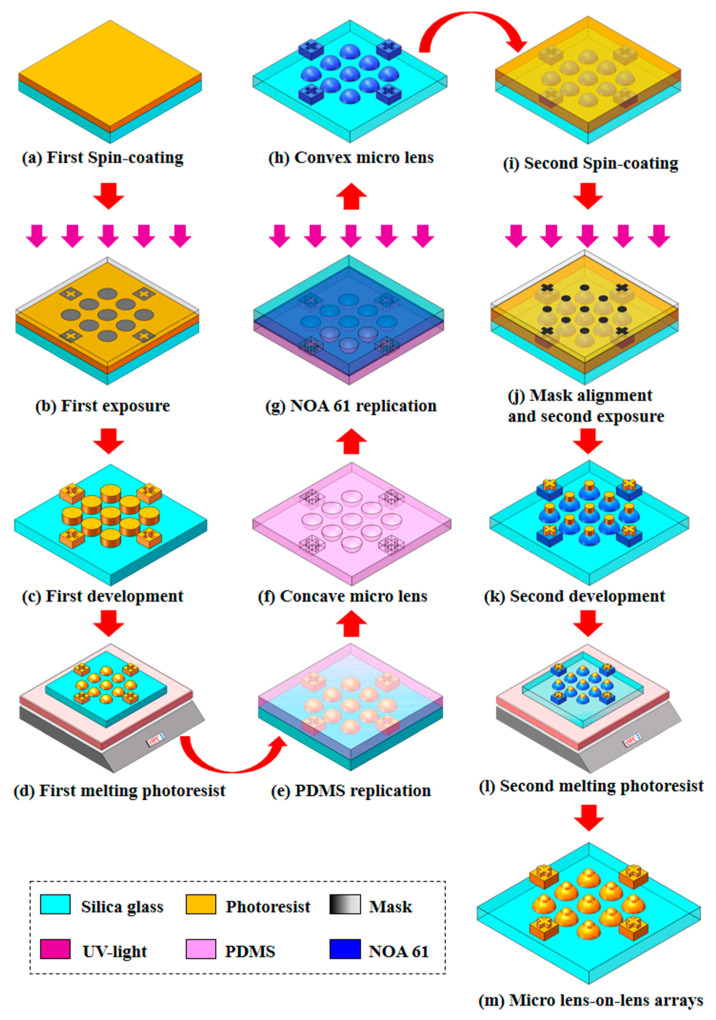
Schematic of the fabrication processes for MLLAs. (**a**–**d**) Fabricated lower-layer microlens arrays using the photoresist reflow method, including spin-coating the photoresist on the silica substrate, first exposure with the first mask, development and photoresist reflow. (**e**,**f**) Replica process of the microlens arrays on PDMS surface by soft lithography technology. (**g**,**h**) Replica process of the microlens arrays on the NOA61 surface using nano-imprint technology. (**i**–**l**) Fabricated upper-layer microlens arrays using mask alignment exposure technology with the second mask and photoresist reflow. (**m**) The fabricated MLLA structure.

This paper proposes a novel approach to fabricate MLLAs based on the traditional photoresist reflow method. MLLAs with high precision can be obtained by introducing soft lithography, nano-imprint technology and mask alignment exposure technology. The fabrication processes are shown in Figure 3.

**Figure 4:**
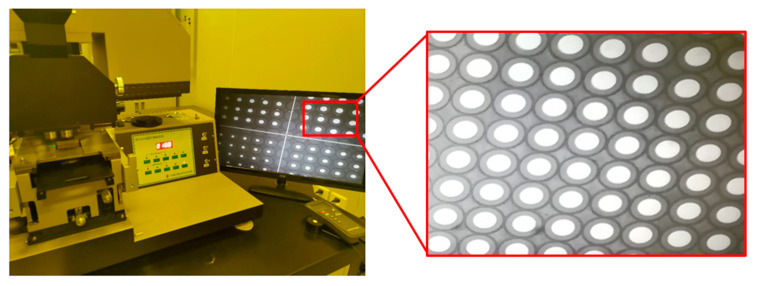
Measured results of the mask alignment by a scanning electron microscope.

First, the first mask pattern is translated onto the photoresist, in which the thickness is *H*_2_ using the contact exposure technology, as shown in Figure 3b. The photoresist cylindrical array with an aperture of *d*_2_ and a period of *T* is formed as shown in Figure 3c. The single layer microlens array with a height of *h*_2_ is fabricated using the traditional photoresist reflow method, as shown in Figure 3d. A concave microlens array is achieved on a polydimethylsiloxane (PDMS) surface using the soft lithography technology, as shown in Figure 3f, and a convex microlens array is obtained on the NOA61 surface using UV nano-imprint technology. The concave and convex microlens arrays are replicated with equal scale to keep them the same size. Mask alignment exposure technology is employed to fabricate the cylindrical array with a thickness of *H*_1_ on the single layer convex microlens array, as shown in Figure 3k. To ensure the fabrication accuracy, the position of the second mask, in which the period is *T* and the aperture is *d*_1_ as shown in Figure 3j, should match the lower-layer structure. Then, the traditional photoresist reflow method is used to fabricate the upper-layer microlens arrays. Figure 4 shows the mask alignment result measured by a scanning electron microscope (SEM) with the magnification of 4×. During this process, the material of the lower layer is NOA61, while the material of the upper layer is photoresist. Finally, dry etching or nano-imprint technology can be used to transform the micro structure onto the substrate to obtain the MLLA structure, as shown in Figure 3m.

In this paper, AZ9260 (520cp) photoresist and AZ400MIF:DI water = 1:1.2 developer are used. The replica processes that are used to maintain the stability of lower-layer microlens arrays and to avoid damage to structures during the follow-up processes contain two parts: the soft lithography technology and the UV nano-imprint technology. The convex lens array on the photoresist is transferred into the concave lens array on the PDMS using the soft lithography technology, and the concave lens array on the PDMS is transferred into the convex lens array on the NOA61 by UV nano-imprint technology. The transmission errors of the morphology are kept under 1% by controlling the temperature. The hardness-Shore D of NOA61 should achieve 85. The mask alignment exposure technology is used to satisfy the high-precision alignment requirement to ensure that the two-layer microlens arrays have the same optical axis, which can affect the image resolution. In this paper, the URE-2000/A contact printer is used as the exposure equipment, and the standard gray-scale mask is used. The required alignment accuracy is better than 0.3 μm.

## 3. Results and Discussion

**Figure 5:**
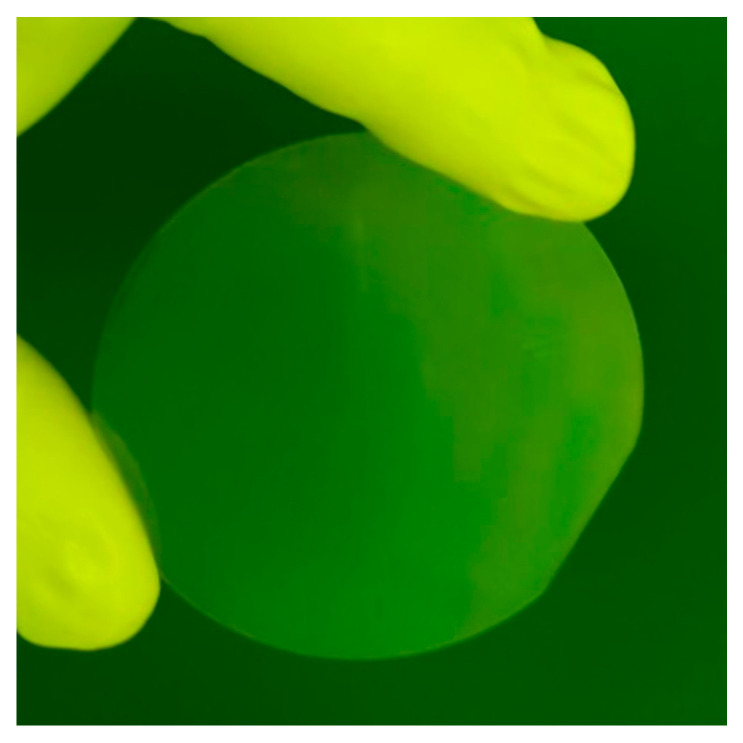
The picture of the MLLA fabricated using our method.

The MLLA is successfully fabricated as displayed in Figure 5. About four hours are taken to fabricate the MLLA using this method. There are hundreds of thousands of micro lens-on-lens structures on the silica substrate, of which the size is two inches, and all of the micro lens-on-lens structures are configured in the shape of quadrangle. Some important parameters of the processing are shown in Table 1. The period of the microstructure (*T*) is 100 μm. The aperture of the mask used in the first exposure process of the lower-layer microlenses (*d*_2_) is 96 μm, while the aperture of the mask used in the second exposure process of the upper-layer microlenses (*d*_1_) is 40 μm.

**Figure 6:**
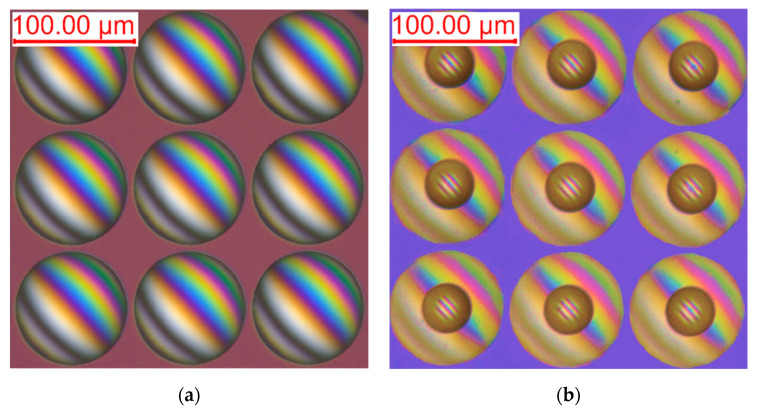
The morphology of the MLLA detected using a differential interference microscope. (**a**) The morphology of microlens array in the lower layer. (**b**) The structure of the MLLA.

The morphology of the MLLA is detected using a differential interference microscope. Figure 6a shows the microlens array in the lower layer. Figure 6b shows the MLLA structure that has two focal lengths. The figures indicate that the MLLA has satisfactory consistence and smooth surfaces without obvious defects. Additionally, the optical axes of the lower-layer microlenses coincide with the upper ones, which is one of the key factors that ensure the image quality. The figures show the perfect uniformity and spherical morphology of the MLLA.

**Figure 7:**
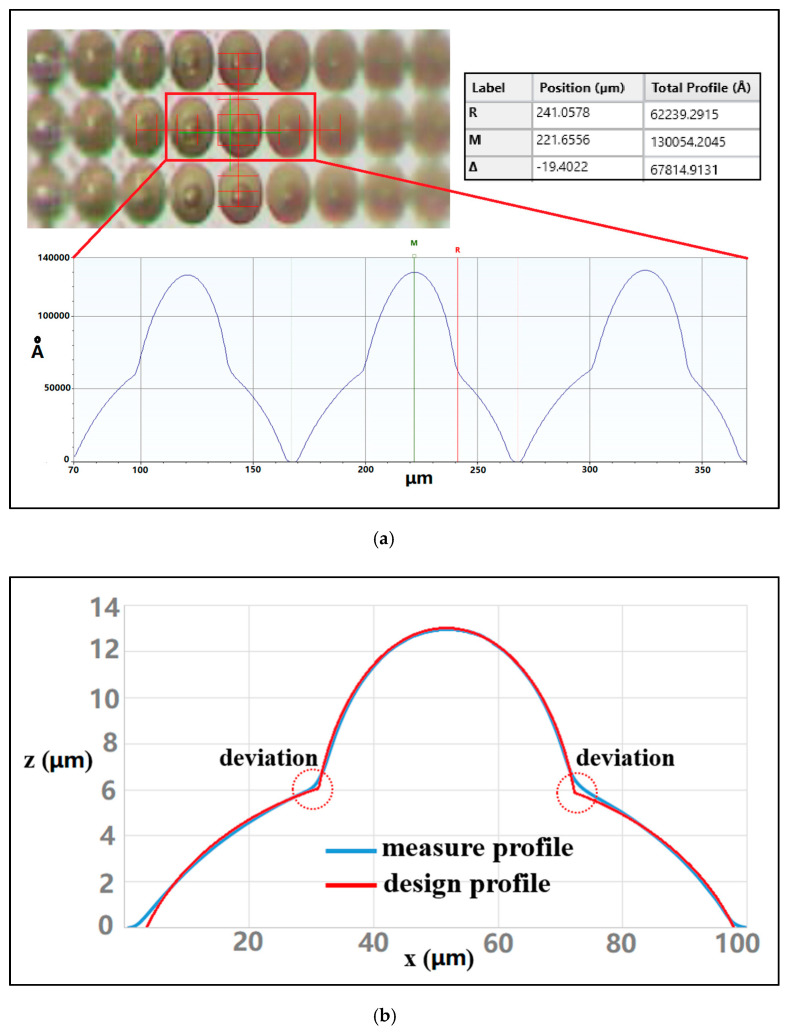
Cross-sectional profiles of the MLLA. (**a**) Cross-sectional profiles of the MLLA. (**b**) Comparison of the designed and measured cross-sectional profiles of the MLLA.

The geometrical morphology parameters of the MLLA are measured using a Dektak XT surface profilometer, and the results are shown in Figure 7a. The height of the lower-layer microlens (*h*_2_) is 6.7 μm, and the height of the upper-layer microlens (*h*_1_) is 6.9 μm. The focal lengths of the microlenses can be calculated according to Equation (3). For the lower-layer microlens, the focal length (*f*_2_) is 344 μm. For the upper-layer microlens, the focal length (*f*_1_) is 58 μm. There are great differences between the two focal lengths, which is beneficial to separate the image planes and to expand the depth of field. Both designed and measured cross-sectional profiles of the MLLA are presented in Figure 7b. The figure indicates that the measured cross-sectional profiles are consistent with the designed ones, with only a little deviation at the upper-lower interface. The average deviation of the morphology is less than 2%, satisfying the application requirements.

**Figure 8:**
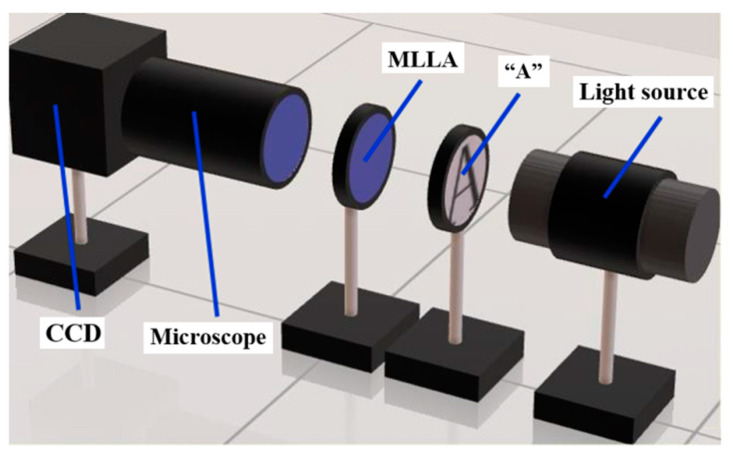
Schematic of the experiment system.

To verify the image quality of the MLLA, an experiment is carried out, as shown in Figure 8. A mask with letter “A” is put between the light source of the microscope and the MLLA, and images are observed by a CCD camera, which is equipped on the microscope.

**Figure 9:**
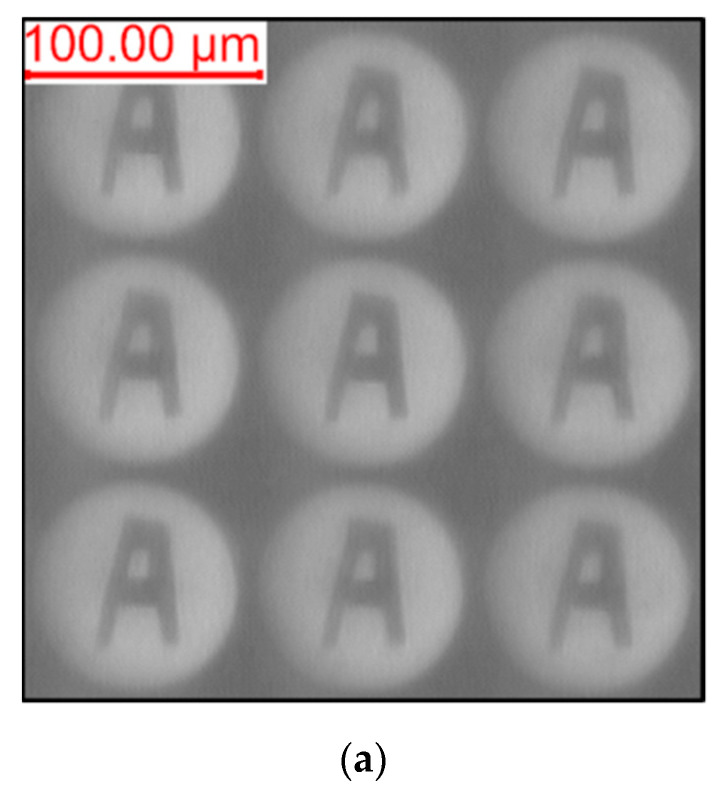

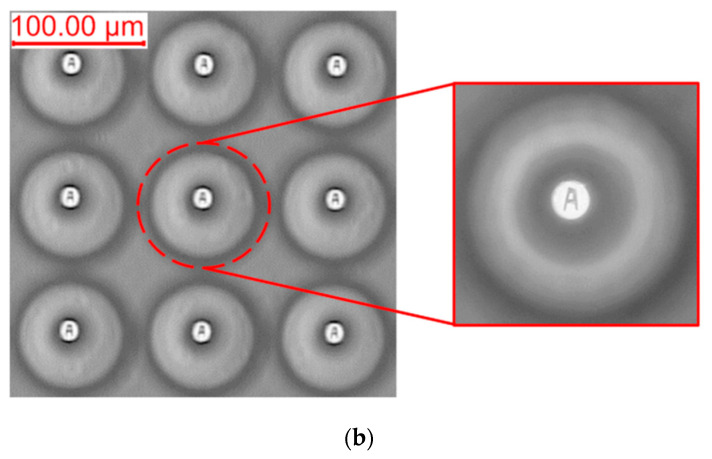
The focusing and imaging abilities of the MLLA. (**a**) Images obtained in the focal plane of the lower-layer microlenses. (**b**) Images obtained in the focal plane of the upper-layer microlenses.

Figure 9 shows the focusing and imaging abilities of the MLLA. Figure 9a shows the images obtained in the focal plane of the lower-layer microlenses, and Figure 9b shows the images obtained in the focal plane of the upper-layer microlenses. As the figure shows, there is a larger magnification for long focal length compared with the short one. However, the sharpness of images is much larger for short focal lengths due to the energy dispersion in the position of long focal plane. The figures demonstrate that the MLLA has an excellent imaging ability, indicating that the MLLA can satisfy the application requirements.

## 4. Conclusions

This paper proposes a novel approach to fabricate MLLAs based on the traditional photoresist reflow method. MLLAs with high precisions can be obtained by introducing soft lithography technology, nano-imprint technology and mask alignment exposure technology. Using this method, MLLAs with different sizes and focal lengths can be fabricated by adjusting mask patterns and processing parameters. MLLAs with focal lengths of 344 μm and 58 μm are fabricated, and the measured profiles are consistent with the designed ones. The average deviation in the morphology is less than 2%, verifying the feasibility and validity of the method. An experiment is carried out to measure the image quality of the MLLA. The results show that clear images can be obtained in the positions of the two focal planes. Both focusing and imaging abilities of the MLLA are confirmed to be excellent, indicating that our approach is an efficient method for fabricating high-precision MLLAs and has an extensive application prospect.

## Figures and Tables

**Table 1:** The parameters of the mask used to fabricate the MLLA.

Microstructure	*T* (μm)	*D* (μm)	*H* (μm)	*H* (μm)	*F* (μm)
1—upper micro lens arrays	100	40	4.5	6.9	58
2—lower micro lens arrays		96	4.7	6.7	344
